# Supplementary material for: Association between oral microbiome and seven types of cancers in East Asian population: a two-sample Mendelian randomization analysis
Source: Front Mol Biosci. 2023 Nov 21;10:1327893. doi: 10.3389/fmolb.2023.1327893 (PMC10702768; doi:10.3389/fmolb.2023.1327893)
Supplement: Supplementary file 1 [file DataSheet1.ZIP › Supplementary Materials/MR plots for tongue/tongue═╝/Pancreatic cancer/╥╚╧┘░⌐_╔╕╤í╜ß╣√/pheno.1762_to_pancreatic cancer_forest.pdf]

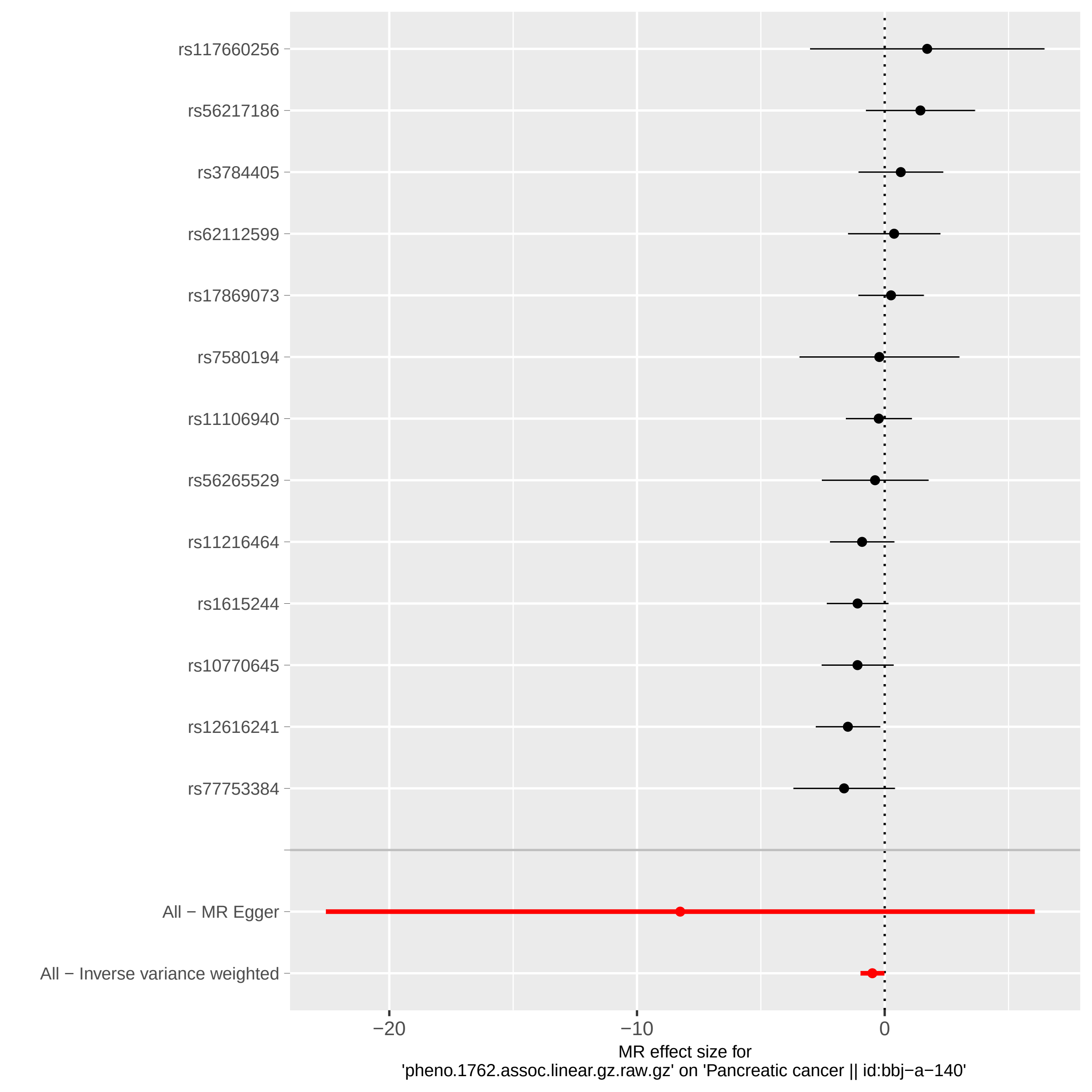

rs117660256
rs56217186
rs3784405
rs62112599
rs17869073
rs7580194
rs11106940
rs56265529
rs11216464
rs1615244
rs10770645
rs12616241
rs77753384
All − MR Egger
All − Inverse variance weighted
−20
−10
0
MR effect size for
'pheno.1762.assoc.linear.gz.raw.gz' on 'Pancreatic cancer || id:bbj−a−140'
